# GAM-NGS: genomic assemblies merger for next generation sequencing

**DOI:** 10.1186/1471-2105-14-S7-S6

**Published:** 2013-04-22

**Authors:** Riccardo Vicedomini, Francesco Vezzi, Simone Scalabrin, Lars Arvestad, Alberto Policriti

**Affiliations:** 1 Department of Mathematics and Computer Science, University of Udine, 33100 Udine, Italy; 2 IGA, Institute of Applied Genomics, 33100 Udine, Italy; 3 KTH Royal Institute of Technology, Science for Life Laboratory, School of Computer Science and Communication, 17121 Solna, Sweden; 4 Swedish e-Science Research Centre, Dept. of Computer Science and Numerical Analysis, Stockholm University, 17121 Solna, Sweden

## Abstract

**Background:**

In recent years more than 20 assemblers have been proposed to tackle the hard task of assembling NGS data. A common heuristic when assembling a genome is to use several assemblers and then select the best assembly according to some criteria. However, recent results clearly show that some assemblers lead to better statistics than others on specific regions but are outperformed on other regions or on different evaluation measures. To limit these problems we developed GAM-NGS (Genomic Assemblies Merger for Next Generation Sequencing), whose primary goal is to merge two or more assemblies in order to enhance contiguity and correctness of both. GAM-NGS does not rely on global alignment: regions of the two assemblies representing the same genomic *locus *(called *blocks*) are identified through reads' alignments and stored in a *weighted *graph. The merging phase is carried out with the help of this weighted graph that allows an *optimal *resolution of *local *problematic regions.

**Results:**

GAM-NGS has been tested on six different datasets and compared to other assembly reconciliation tools. The availability of a reference sequence for three of them allowed us to show how GAM-NGS is a tool able to output an improved reliable set of sequences. GAM-NGS is also a very efficient tool able to merge assemblies using substantially less computational resources than comparable tools. In order to achieve such goals, GAM-NGS avoids global alignment between contigs, making its strategy unique among other *assembly reconciliation *tools.

**Conclusions:**

The difficulty to obtain correct and reliable assemblies using a single assembler is forcing the introduction of new algorithms able to enhance *de novo *assemblies. GAM-NGS is a tool able to merge two or more assemblies in order to improve contiguity and correctness. It can be used on all NGS-based assembly projects and it shows its full potential with multi-library Illumina-based projects. With more than 20 available assemblers it is hard to select the best tool. In this context we propose a tool that improves assemblies (and, as a by-product, perhaps even assemblers) by merging them and selecting the generating that is most likely to be correct.

## Background

The advent of Next Generation Sequencing (NGS) technologies made possible to sequence virtually all the organisms of the biosphere [1]. NGS technologies are characterized by extremely high data production which makes it affordable to obtain high coverage of any organism.

The ability to produce high sequence coverage for lots of genomes paved the way to a large number of *de novo *assembly projects [2,3]. Despite this, it is now commonly accepted that *de novo *assembly with short reads is more difficult than *de novo *assembly with long Sanger reads [4]. Short read length and reduced insert size made correct assembling and positioning of repeats a very crucial and delicate issue. Even though some papers presented high quality assemblies based on NGS data (see [5,6]), *de novo *assembly, especially for large eukaryote genomes, is still a holy grail [7,8].

Recently, several evaluations have been presented, trying to rank assemblers' performances on different datasets: Assemblathon [9] and GAGE [10] are among the most important ones. As a byproduct, these "competitions" showed that it is extremely difficult to establish the best assembler. Each dataset is characterized by different peculiarities and the heuristics implemented by a single assembler are usually only partially able to solve the raised issues.

An interesting strategy to improve *de novo *assemblies has been proposed and goes under the name of *assembly reconciliation *[11,12]. The goal of assembly reconciliation is to merge the assemblies produced by different tools while detecting possible mis-assemblies and isolating problematic regions. Such a strategy has already been proposed for Sanger-based assemblies and one of the goals of this paper is to study its adaptation to NGS data.

Zimin *et al. *in [11] presented Reconciliator, which is based on an iteration of errors identification and correction, and merging phases. Using the so called CE statistics [11] they identify regions likely to contain errors in the assemblies. After this, a global alignment between the two assemblies is performed. In order to avoid problems with repeats, alignment is performed using seeds unique in both the reference and the query sequences. At this point areas marked as problematic are solved using the assembler with better CE statistics and possible gaps in the assemblies are filled. The last step consists in the validation of the merged assembly.

Casagrande and colleagues in [12] proposed GAM (GAM-NGS's ancestor), a tool similar to Reconciliator, but able to avoid the global alignment step. In order to identify similar sequences they searched for areas assembled by the same reads. Subsequently the notion of "block" is introduced to evaluate sensible local alignments and a graph is built to describe global relationships between the two assemblies. When confronted with problematic regions (*e.g*., loops and bifurcations in the graph), GAM uses one of the assemblies as guide.

Both Reconciliator and GAM have advantages/disadvantages on one another (*e.g*., GAM does not need a global alignment while Reconciliator does, however GAM was not able to detect and correct mis-assemblies). Nevertheless, both tools share the limitation that they are tailored for Sanger-based assemblers. As an example, they both need a layout file (usually an afg file) describing for each read the (unique) position where it has been used. In NGS assemblers, such a layout file is provided by a small minority of tools (*e.g*., Velvet, Ray and SUTTA). Moreover, another limit of both tools is the fact that the two input assemblies must have been produced using the *same *set of reads.

Recently, two new tools appeared, tackling the problem of assembly reconciliation using NGS-like datasets: GAA [13] and ZORRO [14]. The former one performs a global alignment between two assemblies (using BLAT). The alignment is used to build the so called Accordance Graph in order to merge the assemblies. In the merging phase reads are used to solve possible inconsistent links in order to output a correct assembly. The latter one, ZORRO [14], performs a first error correction phase directly on the original contigs and then a global alignment using *nucmer*. The alignment is used to order contigs and deriving a consensus sequence. The main drawback of both GAA and ZORRO is the mandatory global alignment phase between the assemblies, which is not only a computational expensive step, but, in presence of ortholog and paralog sequences, it may produce a large number of false links affecting merging performances. Morover, GAA focuses more on avoiding mis-assemblies' introduction than correcting them, while ZORRO is explicitly designed for short genomes (as size increases, merging is not feasible).

Other tools that belong to the assembly reconciliation family are MAIA [15], e-RGA [16], and the Velvet's Columbus module. However, they focus more on enhancing *de novo *assembly results guided by a reference sequence belonging to closely related species, than on pure reconciling *de novo *assemblies.

With this picture in mind we developed GAM-NGS (Genomic Assemblies Merger for Next Generation Sequencing) whose primary goal is to merge two assemblies in order to enhance contiguity and possibly correctness. GAM-NGS does not need global alignment between contigs, making it unique among assembly reconciliation tools. In this way not only a computationally expensive and error prone alignment phase is avoided, but also much more information is used (total read length is usually one or two order of magnitude higher than the mere assembly's length). Read alignments allow the identification of regions reconstructed with the same reads, thus isolating natural candidates to represent the same genomic *locus*. GAM-NGS merge-phase is guided by an Assemblies Graph (AG). AG is a weighted graph and this is another specific feature of our tool. Weights indicate the likelihood that a link is part of a correct path. AG allows GAM-NGS to identify genomic regions in which assemblies contradict each other (loops, bifurcations, *etc*.). In all these situations weights are *locally *used to output the most reliable sequence, given the information in AG.

GAM-NGS requires as input two assemblies and a SAM-compatible alignment (*e.g*., obtained with BWA [17], ERNE [18]) for each input read library and each assembly. GAM-NGS can also work with assemblies obtained using different datasets, as long as the set of reads aligned on the assemblies is the same. It is important to note that, mapping reads back to the assembly is practically a mandatory phase for a large number of downstream analyses (*e.g*., SNP calling, repeat analyses, *etc*.) and therefore represents no extra cost.

We tested GAM-NGS on six datasets. We used three GAGE datasets [10] in order to evaluate GAM-NGS and to compare it with other assembly reconciliators (*i.e*., GAA and ZORRO). Moreover, in order to show GAM-NGS data and "biological" scalability, we tested it on three large plant datasets: a *Prunus persica *genome (227 Mbp, double haploid), a *Populus nigra *genome (423 Mbp, heterozygous) and a *Picea abies *genome (20 Gbp, diploid and highly repetitive). GAM-NGS turned out to be able to correctly merge these assemblies, significantly improving the results achievable using only one assembler. Statistics computed on GAM-NGS outputs show comparable results with respect to other assembly reconciliation tools. Nevertheless, GAM-NGS is always the fastest and the least computationally demanding tool, which makes GAM-NGS the best candidate for large datasets.

## Methods

GAM-NGS's main idea is to identify highly similar fragments between two assemblies, searching for regions sharing a large amount of mapped reads. The assumption is that areas built using the same reads most likely represent the same genomic *locus*.

The vast majority of NGS assemblers does not return a layout file as output (*i.e*., a file, usually in afg format, listing along the assembly the reads used and their positions). In order to overcome this limit, GAM-NGS approximates the layout file using reads aligned back to the assembly: an analysis step almost mandatory in all *de novo *assembly projects. Such an approximation may turn out errors prone: as an example, consider a genome containing (almost) perfectly duplicated regions. In such a case genomic read belonging to any two repeated sequences will be randomly assigned to one of the two copies. In order to keep problems related with repeats, at least partially, under control, GAM-NGS uses only reads aligning to a single position (a.k.a. *uniquely aligned*), discarding all reads that have two or more high scoring alignments (a.k.a. *ambiguously aligned*).

As a matter of fact, since assemblers implement different heuristics (if this was not the case, merging would be trivial), they may contradict each other by inverting sequences' order or erroneously merging (*e.g*., scaffolding) sequences belonging to different genomic regions. Thus, it is compulsory to identify these situations and, possibly, solve them. To address this problem we used a graph structure (dubbed Assemblies Graph or AG) recording and weighting the most probable order relation among regions, *blocks*, where the same reads are mapped.

Once AG is built, GAM-NGS identifies "problematic" regions, signalled by specific sub-graph structures. Such local problems are solved by selecting the path in the graph that maximizes a set of measurable and local features, suggesting the assembly's correctness. Some of these features are borrowed from [19] and are computed using pairing information coming from aligned paired-end and possibly mate-pair reads libraries. If there is not enough evidence to decide on assembly correctness (*e.g*., weights are too close to each other), we chose to be as conservative as possible, electing one of the sequences as *master*, the other one, therefore, becoming the *slave*. In the following sections we will denote the *master *assembly as *M *and the *slave *one as *S*.

After this last phase, GAM-NGS visits the simplified graph, merges contigs finding a consensus sequence and finally outputs the improved assembly.

### Definitions

Let Σ be an alphabet and Σ* be the set of finite-length strings from Σ. For every *s *∈ Σ^* ^we will denote by |*s*| the number of characters in *s*. In our context reads and contigs are elements of Σ*, where Σ = {*A*, *C*, *T*, *G*, *N*}. With $R=\left\{r_{1},r_{2},...,r_{n}\right\}$ we denote the set of reads aligned against both *M *and *S*, which are the master and slave assemblies, respectively. Usually R  is the set, or a subset, of reads used to assemble both *M *and *S *and its elements may belong to different paired read and mate pair libraries. However, alignments of reads belonging to different libraries should be provided into separate alignment files, in order to exploit the information of different inserts' sizes.

Let *r*_1_, *r*_2 _be two reads aligned against the same contig *C *(with *C *belonging to either *M *or *S*). For *i *∈ {1, 2}, let *begin*(*r_i_*) and *end*(*r_i_*) be the positions in *C *where the first and last base of *r_i _*are aligned, respectively. Therefore, we can assume *begin*(*r_i_*) <*end*(*r_i_*), for *i *∈ {1, 2}. We say that *r*_1 _and *r*_2 _are *adjacent *if and only if *begin*(*r*_2_) ≤ *end*(*r*_1_)+1 and *begin*(*r*_1_) ≤ *end*(*r*_2_) + 1.

Given a contig *C *belonging to assembly *A*, a *frame *over *A *is defined as a sequence of reads *r*_1_, ..., *r_n _*mapped against *A *where *r_i_*, *r*_*i*+1 _are adjacent for *i *= 1, ..., *n *- 1. Thus, a frame *F *can be identified by the contig where its reads are aligned and the interval [*begin*(*F*), *end*(*F*)], where *begin*(*F*) = min{*begin*(*r_i_*)|*i *= 1, ..., *n*} and *end*(*F*) = max{*end*(*r_i_*)|*i *= 1, ..., *n*}. Moreover, we define the length of a frame *F *as |*F*| = *end*(*F*) - *begin*(*F*) + 1.

Given two different assemblies *M *and *S*, we define a *block B *as a pair of frames (one over *M *and one over *S*) consisting of the same sequence of reads *r*_1_, ..., *r_n_*, and the size of the block as the number of reads it is composed of. If the majority of the reads *r_i _*are aligned with opposite orientations on the two frames, we say that *B *is *discordant*. Otherwise, we say that *B *is *concordant*. We are interested in finding blocks where the reads' sequence (the frame) is as long as possible. Ideally, blocks should represent those fragments of the considered genome which have been built in accordance by both the assemblies.

In the following we will first explain how blocks are built from alignments and then we will show how blocks are filtered in order to avoid spurious blocks produced as consequence of the existence of similar genomic regions. After this we will illustrate the Assembly Graph construction, the handling of the problematic regions identified on the graph and, lastly, how the merging phase is carried out.

### Blocks construction

The first, and most computational demanding, step of GAM-NGS's outer algorithm is the identification and construction of blocks between assemblies *M *and *S*. The basic input format are BAM files (*i.e*. file in the, by now, standard alignment format). Alignments are assumed to be ordered by their contig identifier and by the alignment position.

The procedure starts by loading into a hash table H  all the reads uniquely mapped on *M *(memorizing only the strictly necessary data). Once H  has been populated, uniquely mapped reads on *S *are processed. In particular, for each read *r*, we perform the following steps:

• if *r *is not present in H , we will not use it for blocks construction;

• if *r *is adjacent to a previously created block *B *(*i.e*., adjacent to a read contained in both its frames), then *B *is extended using *r*;

• otherwise, a new block, started by the single read *r*, is built.

Storing in main memory all the alignments of *M *and going through all the alignments of *S *may easily become a major computational stumbling block. For this reason we carefully designed the data structures and the relative manipulation algorithm. Each uniquely aligned read requires only 21 bytes: 8 bytes for its identifier, 4 bytes for contig's identifier, starting and ending position, and 1 byte for mapping orientation (reverse complemented or original strand). Moreover, we decided to store them in a memory efficient hash table such as Google's *SparseHash *[20], which is characterized by a 2 bits overhead per entry.

For each processed read *r *mapped on a contig *C *of an assembly *A*, we define the *scope *of *r *as the set of blocks whose frame on *C *is adjacent to *r*. We exploit the fact that input alignments are ordered, during the blocks construction phase: if a block *B *is "out of scope" for the current processed read *r *then *B *will not be successively altered. If the size of *B *is higher than a user predefined threshold *B_min _*then *B *is saved into secondary memory and main memory space is released. Otherwise, *B *is discarded. The rationale behind the *B_min _*threshold is that blocks consisting of only few reads are likely to be a consequence of alignment errors or chimeric sequences.

### Blocks filtering

A typical problem common to all assembly reconciliation tools, is that, especially with highly repetitive genomes, it may happen to merge similar regions belonging to different genomic areas (such a problem is also common among *de novo *assemblers*)*. In particular, GAM-NGS may build blocks between regions that attract the same reads only because they are similar (note that perfect genomic repeats are not a problem because in this case reads will be ambiguously aligned). This situation not only complicates Assemblies Graph's structure, but it also suggests the presence of problematic regions (*i.e*., errors) in sequences that are, in fact, correct. To limit this problem, GAM-NGS runs two additional filtering steps before the graph construction: one based on *depth-of-coverage *analysis, and the other one on *block-length *considerations.

More specifically, considering a block *B *with frames *F_M_*, *F_S_*, on *M *and *S*, respectively, GAM-NGS computes for each frame two different types of coverages: a *block coverage BC *and a *global coverage GC*. For instance, considering the frame on the master assembly *F_M_*, let $R_{F_{M}}$ be the set of *all *reads uniquely aligned on *F_M_*, while let be $R_{B_{M}}$the set of reads uniquely aligned on *F_M _*and used as part of block *B*. Clearly, $R_{B_{M}}\subseteq R_{F_{M}}$. Moreover, we define the block coverage of *F_M _*as

$$BC_{F_{M}}=\frac{\underset{r\in R_{B_{M}}}{\sum }\left|r\right|}{\left|F_{M}\right|}.$$

and the global coverage of *F_M _*as

$$GC_{F_{M}}=\frac{\underset{r\in R_{F_{M}}}{\sum }\left|r\right|}{\left|F_{M}\right|}.$$

**Figure 1:**
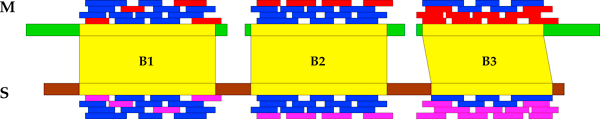
**Blocks construction and coverage filtering**. Blocks are identified by regions belonging to *M *and *S *that share a relatively high amount of mapped reads. In this figure, blue reads identify clusters of adjacent reads that are uniquely mapped in the same contig of both the assemblies. Moreover, GAM-NGS discards blocks like *B*_3 _that contains a small amount of shared reads compared to the number of reads aligned in the same regions (*e.g*., in *B*_3 _these are less than 35% and this block may create a wrong link between contigs).

At this point, GAM-NGS keeps only blocks satisfying the following condition:

$$\text{max}\left\{\frac{BC_{F_{M}}}{GC_{F_{M}}},\frac{BC_{F_{S}}}{GC_{F_{S}}}\right\}\geq T_{c},$$

where *T_c _*is a user defined real number in the interval [0, 1]. The idea is to get rid of blocks built using a low amount of reads compared to the number of mapped reads on both frame intervals (see Figure 1).

We decided to use the maximum between the two ratios in order to avoid the removal of blocks corresponding to heterozygous regions: it may happen that one assembler returns both alleles while the other returns only one of them. In this case, the proportion of reads used in the block should be close to 1 and 0.5, respectively.

The second filtering step is based on the length of block's frames. In particular, given a block *B *composed of frames $F_{M_{i}}$, $F_{S_{j}}$on contigs *M_i _*∈ *M *and *S_j _*∈ *S *respectively, *B *is retained if

$$|F_{M_{i}}|\;\;\geq \text{min}\left\{0.3\cdot \;|M_{i}|,T_{l}\right\}\;\;\vee |F_{S_{j}}|\;\;\geq \text{min}\left\{0.3\cdot \;|S_{j}|,T_{l}\right\},$$

where *T_l _*is a user-defined threshold. Nevertheless, when this condition is not satisfied we still retain the block if any of the following conditions is satisfied: there are other blocks between *M_i _*and *S_j _*satisfying the condition or this is the only block between the two contigs. The rationale is, again, to discard blocks that are likely to be consequences of wrong alignments or chimeric regions, while keeping small blocks that can still witness insertions or deletions by one of the two assemblies.

### Assemblies graph construction

For each assembly, we can define a block order relative to an assembly exploiting frames' order along its contigs. In particular, consider an assembly *A *and two blocks *B*_1 _and *B*_2 _with frames $F_{1}^{A}$ and $F_{2}^{A}$, respectively, both on *A*. We say that *B*_1 _*comes before B*_2 _with respect to *A *if and only if both $F_{1}^{A}$ and $F_{2}^{A}$ lie on the same contig *C_A _*and $F_{1}^{A}$ comes before $F_{2}^{A}$ (*i.e*., *begin*($F_{1}^{A}$) <*begin*($F_{2}^{A}$)) and there is no frame $F_{3}^{A}$ lying over *C_A _*for which $F_{1}^{A}$ comes before $F_{3}^{A}$ and $F_{3}^{A}$ comes before $F_{2}^{A}$.

It is important to point out that this block order strictly depends on the considered assembly, since the same genomic region may have been reconstructed on opposite strands in the input assemblies. Thus, there may be cases where *B*_1 _comes before *B*_2 _with respect to *M*, but *B*_2 _comes before *B*_1 _with respect to *S*. In this scenario, block orders of the two assemblies may contradict each other (leading to cycles in AG) even when there is no contradiction at all.

Our goal is to determine a consistent order of blocks among each contig of both the assemblies. To facilitate that, we build a *Contigs Graph *(CG) which consists of a vertex $V_{M_{i}}$ for each contig *M_i _*∈ *M *and a vertex $V_{S_{j}}$ for each contig *S_j _*∈ *S*. Two vertices *V_U _*and *V_W _*are connected by an undirected edge if and only if *U *and *W *belong to different assemblies and have at least one block over them.

For each edge *e *connecting two vertices $V_{M_{i}}$, $V_{S_{j}}$, we assign the weight

$$w_{e}=\text{max}\left(\frac{r^{+}}{r^{+}+r^{-}},\frac{r^{-}}{r^{+}+r^{-}}\right),$$

where *r*^+ ^and *r*^- ^are the number of reads belonging to concordant and discordant blocks between *M_i _*and *S_j_*, respectively. For each vertex *V *the weight *w_V _*is then computed, corresponding to the mean of its incident edges' weights (this mean is weighted on the overall size of all blocks connecting two contigs). The main idea is that edges' weights will have a value close to one when the majority of the reads composing the blocks are mapped either with the same orientation or with the opposite orientation. In the former case contigs will most likely have the same orientation, while in the latter case one of the two contigs must be complemented and reversed.

In more detail, let Q  be the set of processed vertices. At first, for each connected component of CG, we insert into Q  a vertex *V *which maximizes *w_V _*and we set the original blocks' order for *V*'s contig. Then, we repeat the following steps until all vertices of the graph belong to Q :

• Pick V∈Q with largest *w_V_*;

• Let *adj*(*V*) be the set of the vertexes adjacent to *V*. For each vertex *V_U _*∈ *adj*(*V*), we set the order of blocks on *U *depending on whether the majority of reads belongs to concordant or discordant blocks and according to blocks' order of *V*'s contig;

• *adj*(*V*)'s elements are added to Q  and we remove *V*'s incident edges from the graph, updating vertices' weights.

The rationale behind this heuristic is that, at each iteration, we set the order of the blocks over one of the contigs for which we have the clearest evidence. However, this is a simple (yet effective) procedure to compute a consistent blocks' order among the assemblies and we plan to improve it in order to have a higher guarantee of avoiding the introduction of "false contradictions" (*i.e*., cycles) in AG.

**Figure 2:**
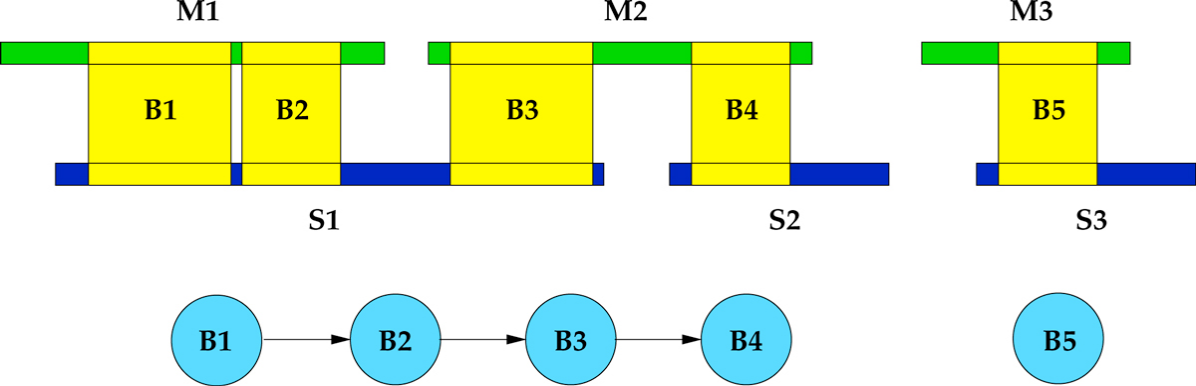
**Assemblies Graph construction**. A simple example of AG construction: *B*_1 _comes before *B*_2 _in both *S*_1 _and *M*_1 _so a directed edge connects $V_{B_{1}}$ and $V_{B_{2}}$. The same also applies for $V_{B_{2}}$ and $V_{B_{3}}$, since *B*_2 _comes before *B*_3 _in *S*_1_. Moreover, an edge is added between $V_{B_{3}}$ and $V_{B_{4}}$ as *B*_3 _comes before *B*_4 _in *M*_2_.

With the updated blocks order we are now able to build the *Assemblies Graph *(AG): a node *V_B _*is added for each block *B*, while edges connect blocks that share at least one frame on the same contig. In particular, if a block *B*_1 _comes before a block *B*_2 _with respect to *M *or *S *we put a directed edge from $V_{B_{1}}$ to $V_{B_{2}}$ (see Figure 2). Notice that, since we are considering the merging of two assemblies, each node cannot have an input or output degree strictly greater than two.

Moreover, during AG construction, we add to each edge a weight characterized by a series of features that are evaluated within the region relative to the blocks related to the vertices connected by the edge.

Let $V_{B_{1}}$, $V_{B_{2}}$ be two nodes linked by an edge (*i.e*., *B*_1 _comes before *B*_2 _on a contig *C *of either one of *M *and *S*). Let *F*_1 _and *F*_2 _be, respectively, their frames on *C*. Then, we compute the number of reads that have a correctly placed pair (or mate) that spans the gap between *F*_1 _and *F*_2 _and the number of reads that are expected to have their pair (or mate) correctly placed and crossing over *F*_1 _and *F*_2 _which is unmapped or mapped to a different sequence. In particular, a read *r*', mapped on a contig *C*, has a correctly placed pair (or mate) *r*" if *begin*(*r*") is inside the region [*begin*(*r*') + (*m *- 3 · *sd*), *begin*(*r*') + (*m *+ 3 · *sd*)] and |*C*| ≥ *begin*(*r*') + (*m *+ 3 · *sd*), where *m *and *sd *are the mean and the standard deviation of the insert size of the library, respectively. Furthermore, we also compute values such as coverage and number of wrongly oriented pairs (or mates). These weights are used to determine the likelihood that a link represents a correct path allowing us to take motivated decisions in case of problematic regions witnessed by non-linear graphs (*i.e*., bubbles, bifurcations, *etc*.).

Every path in AG corresponds to a sequence of blocks such that every pair of consecutive blocks lies on the same assembled sequence for at least one assembly. Thus, we can exploit AG to integrate or extend contigs.

Also, it is important to notice that if we consider AG disregarding edges' orientation, more than a single connected component can be present. We exploited this fact implementing GAM-NGS in a way that it can correct and merge contigs handling single connected components in parallel.

### Handling problematic regions

Even if we build AG using the previously described method, block orders suggested by assemblies may contradict each other. For instance, suppose two blocks lie on a single contig in both the assemblies with opposite order with respect to *M *and *S*. This scenario will lead to a cycle in AG. Moreover, strongly connected components (SCC) containing at least two nodes denote a situation where *M *and *S *disagree on the order of some blocks. To find these kind of contradictions we used Tarjan's algorithm [21] to determine SCC in linear time while visiting AG.

Another possible problem is represented by divergent paths that may indicate situations where assemblies locally behaved differently: one assembler extended a sequence in a different way with respect to the other. In particular, we can exploit edges' weights to perform choices that are locally optimal (*e.g*., in the presence of a bifurcation the path minimizing the evidence of mis-assemblies will be chosen) in order to output a correct sequence. In situations where weights/features do not allow us to take a position (*e.g*. similar weights), we decided to be as conservative as possible, trusting only contigs belonging to the master assembly.

**Figure 3:**
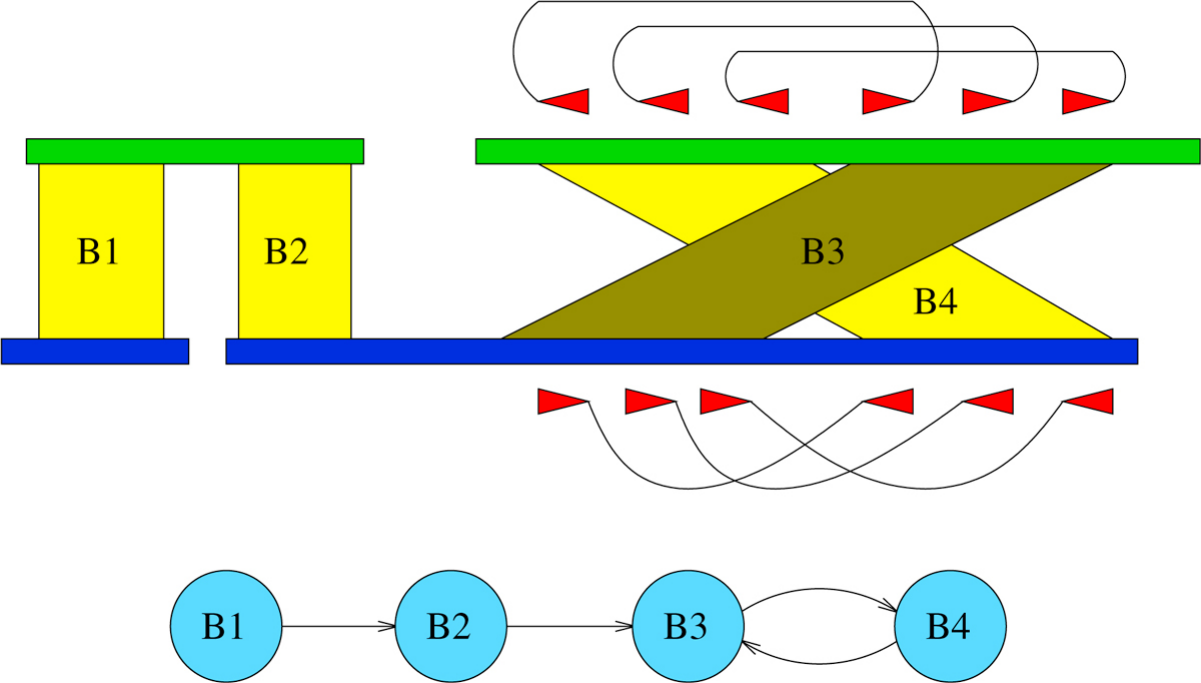
**Handling 2-node cycles in Assemblies Graph**. A 2-node cycle in AG witness a putative inversion along a single contig in *M *and *S*. If there actually is an inversion, then mate-pair reads are aligned with the wrong orientation in one of the two contigs. We can use this information to provide in output a correct sequence (the blue one in the picture).

**Figure 4:**
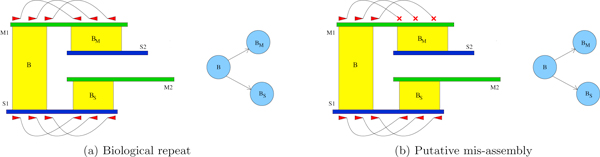
**Handling bifurcations in Assemblies Graph**. Bifurcations in AG, may spot biological repeats or mis-assemblies. In panel (a), paired reads do not solve the bifurcation and we might face a biological repeat. In panel (b), paired reads on *M*_1 _might help us to spot a mis-join in the assembly.

Among the various graph structures generated by discordant assemblies, *bubbles *and *forks *are the most common ones (see Figures 3 and Figure 4). Bubbles consist of a path that first diverges and then converges back. Forks, instead, contain only divergent or convergent paths. We can spot and distinguish these two structures with a simple depth-first traversal of AG. Such structures can nest in highly complex scenarios and, at this stage, we decided to deal only with graphs for which we have a good guarantee that they will be handled correctly. In particular, we took care only of cycles involving exactly two nodes and bifurcations not involving any bubble.

#### Handling cycles involving exactly two nodes

Cycles involving only two nodes may indicate inversions along the same contig in both *M *and *S*. To solve this particular kind of loop we can exploit mate-pair and pair-end reads' orientation. In [19] it has been shown how the use of mate-pair-happiness [22] is one of the best methodologies to detect mis-assemblies.

If the graph is indeed the result of two inverted blocks in one of the two assemblies, contigs pairs will be mapped with the correct orientation in only one of the two (see Figure 3). Hence, if we are able to find a minimum number of reads that are aligned properly in one contig and with the wrong orientation in the other one, we can include the correct sequence in the improved assembly. Otherwise, we chose to directly output the sequence of the master assembly.

#### Handling bifurcations

Graphs containing bifurcations may signify biological repeats or mis-assemblies. We will only show how we handle nodes with output degree equal to two, since nodes with input degree equal to two can be treated symmetrically. Let *B *be a block such that *V_B _*has two outgoing edges to $V_{B_{M}}$ and $V_{B_{S}}$. Let *M_i _*∈ *M *be the contig shared between *B *and *B_M_*, and *S_j _*∈ *S *be the contig between *B *and *B_S_*. In order to solve this scenario we focus on where reads placed on frames defined by B have their respective paired read (or mate): do they end up in *B_M _*or *B_S_*? See Figure 4 for an illustration of this case. Let *n_M _*and *n_S _*count the number of mates mapped to *B_M_*'s and *B_S_*'s frame, respectively. Given a read library with mean insert size *m *and standard deviation *s*, we define *u_M _*(respectively *u_S_*) as the number of reads mapped on the frame defined by *B *such that their pair/mate, accordingly to library orientation, is *not *aligned within a region of length *m *+ 3 · *s *(*i.e*., insert size spanning) in *B_M_*'s frame on *M_i _*(respectively, in *B_S_*'s frame on *S_j_*). If *M_i _*(or *S_j_*) is so short that it is included within the insert size spanning of a read placement, then that read is not used to compute *u_M _*(or *u_S_*).

For instance, if we find that

$$\frac{n_{M}}{u_{M}}\geq T_{U}\;\wedge \;\;\frac{n_{S}}{u_{S}}\leq T_{L},$$

where *T_U _*>*T_L _*are two threshold values in [0, 1], we may be able to spot a mis-assembly in *S_j_*. Conversely, if we find that

$$\frac{n_{S}}{u_{S}}\geq T_{U}\;\;\wedge \;\;\frac{n_{M}}{u_{M}}\leq T_{L},$$

we may be able to spot a mis-assembly in *M_i_*, as in Figure 4(b). If we are not in any of the two previous situations, it might mean that either blocks are too distant to let us discover the mis-assembly or *B *has been built due to a repetitive sequence. In this case, to avoid the introduction of errors in the improved assembly, we do not risk resolving the bifurcation and instead simply output the master's contigs.

### Merging

After solving problematic regions in AG, we can visit maximal disjoint paths in order to produce a draft alignment of contigs belonging to different assemblies. Such alignment is based on reads mapping and might be inaccurate (*e.g*., regions having low identity). Therefore, we perform a semi-global alignment algorithm [23] (a banded variant to save memory) to make sure that contigs have a high similarity (*i.e*., at least an identity of 95%) and should be merged.

We decided *not *to return a consensus, since there is no guarantee that it would be better than the two original sequences. Therefore, we decided to output the sequence belonging to the assembly that locally shows the best CE statistics [11] for insert sizes.

**Figure 5:**
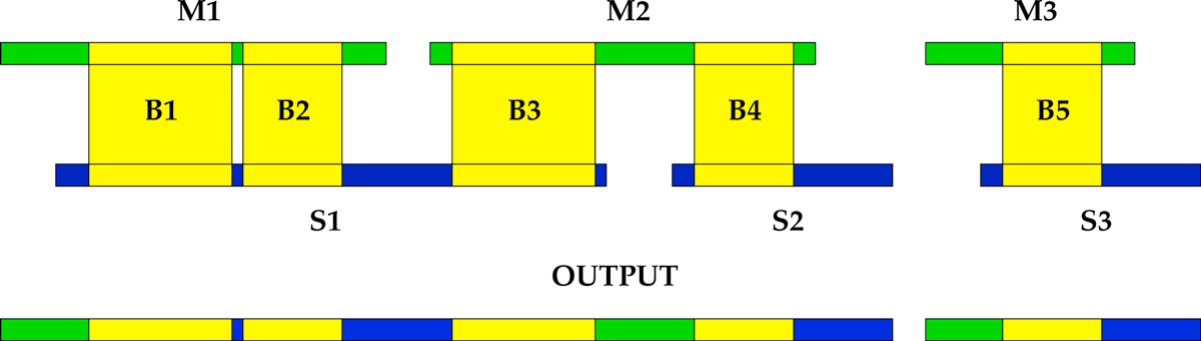
**GAM-NGS's merging phase**. During the merging phase, we fill the gaps between contigs in *M *and we extend a contig of *M *only if the corresponding sequence in *S *is longer and semi-global alignments at any end do not drop below 95% identity. Moreover, for regions defined by a block, we output the frame with better CE statistics.

We also tried to avoid the introduction of duplicated regions, closing a gap between two contigs of *M *linked by a contig of *S *if and only if semi-global alignments on both ends of the region do not drop below 95% identity (see Figure 5).

After this phase, we obtain a set of *merged *contigs that we called *paired contigs*. To obtain the final improved assembly we simply output this set along with contigs of *M *that were not involved in any merge.

## Results and discussion

Validation of GAM-NGS's output has been performed on public data, for which results obtained by various assemblers are public as well. In particular, we chose three real datasets (*i.e*., *Staphylococcus aureus, Rhodobacter sphaeroides *and human chromosome 14) downloaded from GAGE [10] website [24] (see Table 1) for which a reference genome is available. Moreover, we chose to test GAM-NGS on larger datasets such as *Prunus persica*, *Populus nigra *and *Picea abies*, in order to show our tool's scalability.

**Table 1:** Reference genomes and libraries for public datasets (Allpaths-LG corrected)

Organism	Genome length (bp)	Library	Avg Read length (bp)	Insert size (bp)	Coverage
*S. aureus*	2,903,081	Fragment	101	180	29X
		
		Short jump	96	3500	32X

*R. sphaeroides*	4,603,060	Fragment	101	180	31X
		
		Short jump	101	3500	29X

Human chr14	88,289,540	Fragment	101	180	39X
		
		Short jump	96	3000	12X
		
		Long jump	96	35000	0.2X

It is also important to point out that datasets provided by GAGE represent a useful instrument to evaluate GAM-NGS for a number of different reasons. First, GAGE provides state of the art datasets formed by several paired end and mate pairs libraries. Second, it provides highly reliable reference assemblies suitable for benchmarking. Third, a suite of reusable scripts is available for computing assembly metrics.

Reads available for each public dataset were error-corrected using both Quake and the Allpaths-LG error corrector. We chose to use the Allpaths-LG error-corrected reads.

Since GAM-NGS (as well as GAA) follows a master/slave approach and many assemblies are available for each GAGE datasets, we had to decide which assemblies should be merged and which should be elected as master.

Evaluating *de novo *assemblies in absence of a reference sequence is as difficult as *de novo *assembly itself. As an example, consider that Assemblathon 2 [25] required more than a year to evaluate submitted assemblies. GAGE datasets gave us the possibility to choose the two best assemblies accordingly to GAGE evaluation, however we decided to be as realistic as possible and to avoid the use of the available reference sequence. To the best of our knowledge, the only methodology available to evaluate assemblies in absence either of a reference sequence or of external-validation-data (*e.g*., fosmid ends, physical maps, *etc*.) is based on Feature Response Curve-analysis (FRCurve-analysis) [19]. Recently, a novel tool dubbed *FRC^bam ^*[26], designed for computing a FRCurve from NGS-datasets, has been presented. Results summarized in [26] show that *FRC^bam ^*is able to effectively detect mis-assemblies. *FRC^bam ^*enabled us to evaluate a *de novo *assembly using only an alignment file (given in SAM/BAM format) of a set of reads (usually the same reads used in the assembly), which is also the same input required by GAM-NGS.

For each GAGE dataset we plotted the FRCurve [19] using *FRC^bam ^*. Then we chose to merge the two assemblies having the steepest curves (*i.e*., few negative features in the longest contigs) and whole length close to the genome size. As expected by the results shown in [26], we were always able to choose assemblies that, using GAGE's evaluation scripts, were characterized by good statistics such as number of errors and corrected NG50 (*i.e*., NG50 of the assemblies broken in correspondence of each mis-assembly). All experiments were performed using both combinations of master/slave assemblies. We also decided to follow a common "bad practice" electing as best assemblies those characterized by the longest NG50 (without any consideration on the number of errors) and run GAM, GAA and ZORRO to merge them.

As far as the three larger datasets were concerned, we merged assemblies obtained with CLC [27] and ABySS [28] for *Prunus persica *and *Populus nigra*, while we used GAM-NGS with a whole genome shotgun assembly and a series of fosmid-pools assemblies (all assembled with CLC assembler) for *Picea abies *that, to the best of our knowledge, represents the largest ever sequenced genome.

GAM-NGS's performance rely on the choice of several parameters: the minimum number of reads per block *B_min_*, the threshold *T_c _*related to blocks' coverage filtering, the minimum block's length threshold *T_l_*.

Low values of *B_min _*increase the number of blocks which leads to a larger memory requirement and to a potentially more complex Assemblies Graph. Moreover, high values of *T_c _*or *T_l _*allow us to filter more blocks, running the risk of discarding significant blocks, while with low values we might keep blocks due to repeats that will complicate AG's structure. We decided to set *B_min _*= 10, *T_c _*= 0.75 and *T_l _*= 200 bp for all experiments on bacteria. Instead, for human chr14, we set *B_min _*= 50, *T_c _*= 0.75 and *T_l _*= 500 bp.

To evaluate correctness, we computed statistics using the same analysis script used in [10] and available for downloading at [24]. In particular, N50 sizes were computed based on the known size of the genome (NG50) and only contigs longer than 200 bp were used for the computations. As a consequence of the absence of a reference sequence in the case of the three new plants genomes we simply returned statistics showing the improvements in contiguity.

All experiments were performed on a 16 CPU machine with 128 GB of RAM, with the only exception of *Picea abies *where we used a machine equipped with 32 CPUs and 2 TB of RAM. A brief description of the commands we used to carry out the merging on all the datasets can be found as supplementary material (see Additional file 1). GAM-NGS was always executed taking advantage of all available CPUs. GAA and ZORRO are designed as single-core programs. For this reason, we reported both CPU and wall clock times for each experiment. Moreover, GAA's internal call to BLAT is specified with the parameter -fastMap which requires input sequences to have contigs shorter than 5 Kbp. Thus, in each experiment, we had to manually run BLAT, providing its output to GAA's call. As we will show later, GAM-NGS was the fastest tool on the largest GAGE dataset (human chromosome 14).

Time of alignment was added to GAM-NGS' time but we would like to emphasize that read alignment is often required in downstream analyses and is also needed when *FRC^bam ^*[26] is used to evaluate assemblies' correctness.

### Evaluation and validation on GAGE datasets

Given the availability of a reference sequence, GAGE datasets allowed us to compute the actual number of errors within an assembly. We compared GAM-NGS with GAA [13] and ZORRO [14] in order to obtain a comparison of assembly reconciliation tools as fair as possible and we used the same scripts used by Salzberg and colleagues in [10], downloadable from [24].

#### Staphylococcus aureus

**Figure 6:**
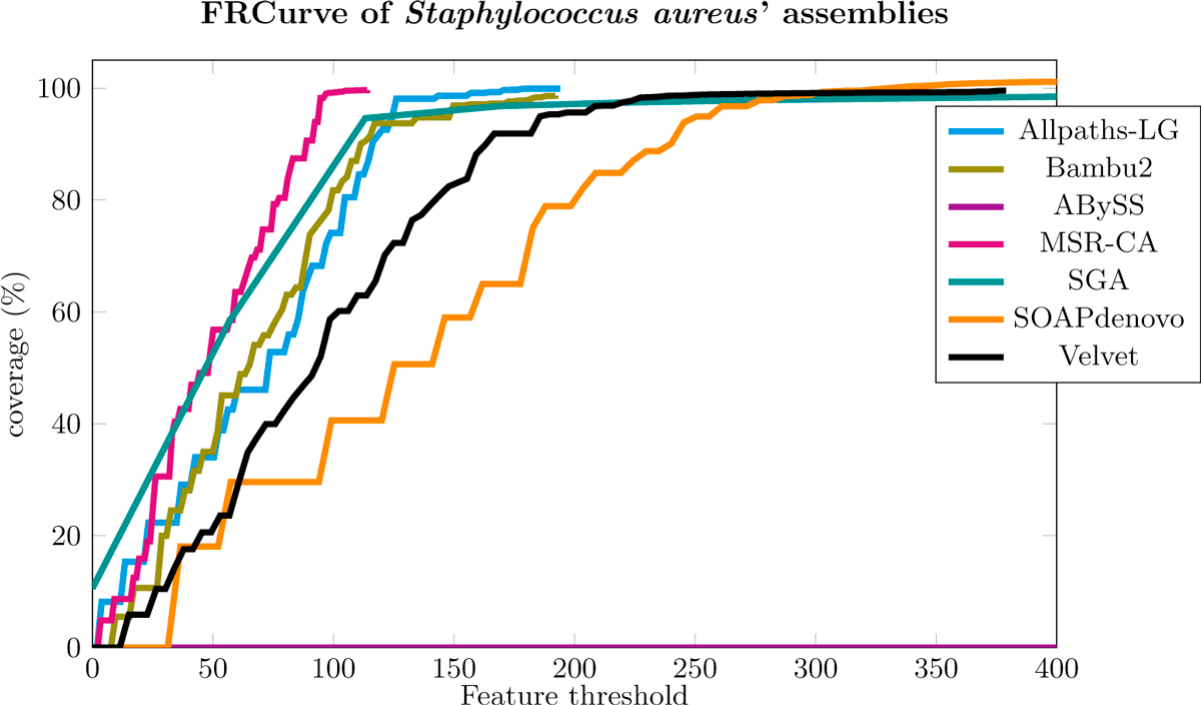
**FRCurve of Staphylococcus aureus' assemblies**. Allpaths-LG and MSR-CA assemblies reach earlier a coverage close to 100% with the smallest number of features and, thus, they where chosen to be merged.

For *Staphylococcus aureus*' dataset we chose to merge the assemblies of Allpaths-LG and MSR-CA. Looking at their FRCurves in Figure 6, they seem to be the best two assemblies for this dataset (SGA looks steeper, however its short contigs contains many issues according to our analysis). This situation is also confirmed by GAGE analysis, as both Allpaths-LG and MSR-CA assemblies have a low number of errors and a large corrected NG50.

As shown in Table 2, using Allpaths-LG as master assembly, GAM-NGS was able to increase Allpaths-LG's NG50 by ~40 Kbp and to decrease the number of compressed regions. Table 2 shows us that GAA behaved better as far as compressed reference bases and corrected NG50 are concerned (GAA's corrected NG50 is ~5Kbp longer than GAM-NGS one). However, GAA is affected by duplication events and, more importantly, Table 3 shows that it contains one misjoin more than GAM-NGS. ZORRO, instead, returned a lower NG50 (about half, compared to GAM-NGS and GAA) and a lower corrected NG50. Moreover, ZORRO's output contains more misjoins than GAM-NGS.

**Table 2:** GAGE statistics (contiguity, duplication and compression) on Staphylococcus aureus.

Assembler	Ctg num	NG50 (kb)	NG50 corr. (kb)	Assembly size (%)	Chaff size (%)	Unaligned ref (%)	Unaligned asm (%)	Dupl (%)	Comp (%)
Allpaths-LG	60	96.74	66.23	98.88	0.03	0.61	0.01	0.04	1.26
MSR-CA	94	59.15	48.23	98.60	0.01	1.28	0.00	0.71	0.88

*Allpaths-LG + MSR-CA*									
**GAM-NGS**	44	141.54	75.82	100.49	0.00	0.44	0.01	0.26	0.99
GAA	40	139.48	80.68	99.52	0.03	0.37	0.01	0.32	0.88
ZORRO	81	74.68	62.85	99.70	0.16	0.32	0.04	0.59	0.88

*MSR-CA + Allpaths-LG*									
**GAM-NGS**	66	90.47	66.44	100.21	0.01	1.01	0.00	2.03	0.89
GAA	53	131.65	64.43	100.66	0.01	0.95	0.00	1.90	0.79
ZORRO	80	74.64	62.85	99.63	0.14	0.32	0.05	0.53	1.11

									

Allpaths-LG	60	96.74	66.23	98.88	0.03	0.61	0.01	0.04	1.26
SOAPdenovo	107	288.18	62.68	100.55	0.34	0.22	0.02	1.66	1.45

*Allpaths-LG + SOAPdenovo*									
**GAM-NGS**	56	107.12	69.39	99.52	0.03	0.56	0.01	0.34	1.26
GAA	40	255.66	83.67	108.10	0.06	0.25	0.01	2.78	1.31
ZORRO	104	76.94	65.83	105.59	0.31	0.15	0.10	5.19	1.36

*SOAPdenovo + Allpaths-LG*									
**GAM-NGS**	93	288.18	62.68	100.92	0.32	0.20	0.02	1.88	1.40
GAA	74	294.96	62.87	101.92	0.34	0.16	0.02	2.62	1.37
ZORRO	107	76.94	62.68	105.63	0.29	0.16	0.09	5.17	1.50

For each assembler we report the number of contigs greater than 200 bp (Ctg), the NG50, the corrected NG50 (NG50 computed breaking the assembly at each error), assembly's total length, the percentage of short (Chaff) contigs, the length of reference's regions which cannot be found in the assembly (Unaligned ref), the length of assembly's regions that cannot be found in the reference (Unaligned asm), the percentage of duplicated (Dupl) and compressed (Comp) regions in the assembly. All the percentages in the table are computed with respect to the true genome size.

**Table 3:** GAGE statistics (SNPs, indels and misjoins) on Staphylococcus aureus.

Assembler	SNPs	Indels < 5 bp	Indels ≥ 5 bp	Misjoins	Inv	Reloc
Allpaths-LG	79	4	12	4	0	4
MSR-CA	191	23	10	13	6	7

*Allpaths-LG + MSR-CA*						
**GAM-NGS**	137	9	15	5	0	5
GAA	145	8	16	6	0	6
ZORRO	133	12	8	6	2	4

*MSR-CA + Allpaths-LG*						
**GAM-NGS**	214	19	10	9	2	7
GAA	206	22	15	11	2	9
ZORRO	262	24	9	7	4	3

						

Allpaths-LG	79	4	12	4	0	4
SOAPdenovo	247	25	31	15	1	14

*Allpaths-LG + SOAPdenovo*						
**GAM-NGS**	88	5	14	4	0	4
GAA	100	9	19	10	1	9
ZORRO	227	19	12	6	1	5

*SOAPdenovo+ Allpaths-LG*						
**GAM-NGS**	304	27	29	15	1	14
GAA	314	32	30	12	1	11
ZORRO	299	28	11	13	2	11

For each assembly we show the number of SNPs, the number of indels shorter than 5 bp and greater (or equal) than 5 bp. The number of misjoins is computed as the sum of inversions (parts of contigs reversed with respect to the reference genome) and relocations (rearrangements moving a contig within/between chromosomes).

Using MSR-CA in place of Allpaths-LG as master assembly, GAM-NGS was able to increase NG50 by ~30 Kbp and provide a better corrected NG50 with respect to the other tools. Moreover, GAM-NGS was able to correct the master assembly problematic regions, as GAM-NGS output has a lower number of misjoins than MSR-CA. GAA, instead, using MSR-CA as master assembly, performed better as far as compressed reference bases are concerned but returned a higher number of misjoins and indels compared to GAM-NGS. In this case ZORRO returned the minimum number of misjoins among the three tools but it is also the one with the assembly characterized by the lowest NG50 and the lowest corrected NG50.

In Table 2 we summarize the results of merging the assemblies characterized by the largest NG50 (*i.e*., Allpaths-LG and SOAPdenovo), without considering assemblies' correctness. The purpose of this test is to demonstrate how important the input assembly choice is. In particular, when using SOAPdenovo as master (*i.e*., assembly with largest NG50) and Allpaths-LG as slave, all the three assemblies reconciliation tools return an assembly characterized by a corrected NG50 lower than master's one. Using Allpaths-LG as master, GAA and ZORRO returned a large number of duplicated regions (providing an assembly much longer than the reference) and they both introduced more misjoins than GAM-NGS.

Table 4 shows running times of the three assembly reconciliation tools. If we consider the CPU time, then GAM-NGS is definitely affected by the required reads alignment phase. Instead, if we consider wall time, GAM-NGS's performance is in line with the other tools.

**Table 4:** Assembly reconciliation tools performances on Staphylococcus aureus.

Tool	User (CPU) time	Wall clock time
*Allpaths-LG + MSR-CA*		
**GAM-NGS**	1h 10' 19" + 51"	4' 10" + 17"
GAA	1' 20"	1' 20"
ZORRO	3' 04"	3' 04"

*MSR-CA + Allpaths-LG*		
**GAM-NGS**	1h 10' 19" + 49"	4' 10" + 17"
GAA	1' 11"	1' 11"
ZORRO	14' 18"	14' 18"

*Allpaths-LG + SOAPdenovo*		
**GAM-NGS**	1h 10' 53" + 33"	5' 12" + 24"
GAA	5' 04"	5' 04"
ZORRO	7' 08"	7' 08"

*SOAPdenovo + Allpaths-LG*		
**GAM-NGS**	1h 10' 53" + 34"	5' 12" + 25"
GAA	4' 49"	4' 49"
ZORRO	9' 52"	9' 52"

In GAM-NGS's entries the first value indicates the time spent in alignment phase, while the second one is GAM-NGS's run time.

#### Rhodobacter sphaeroides

**Figure 7:**
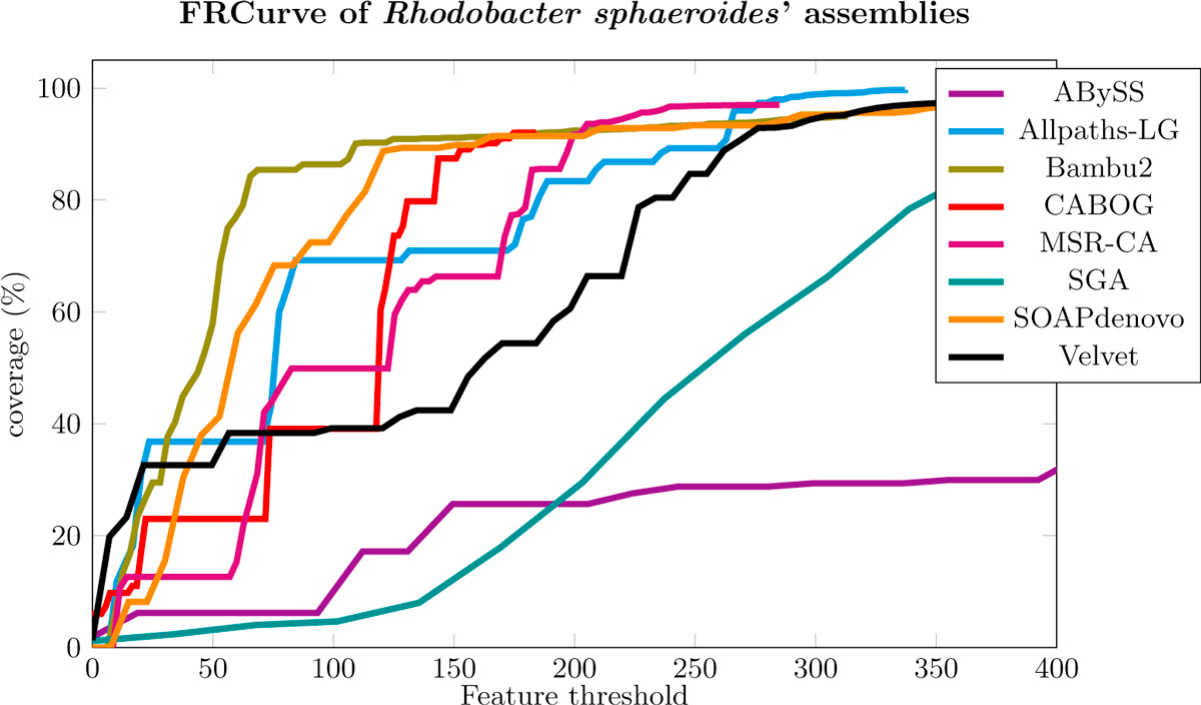
**FRCurve of Rhodobacter sphaeroides' assemblies**. Allpaths-LG and MSR-CA assemblies reach earlier a coverage close to 100% with the smallest number of features and, thus, they where chosen to be merged. CABOG's assembly seems better but provides a low coverage of the genome and, for this reason, it was not taken into account.

For *Rhodobacter sphaeroides*' dataset we chose to merge Allpaths-LG and MSR-CA assemblies. Looking at their FRCurves in Figure 7, they seem the best two assemblies to be merged. CABOG and Bambus2 also provide sharp FRCurves on this dataset, however both assemblies are characterized by a large number of short contigs with many features (*i.e*., long tail), and they both fail to fully assemble the genome, as the total assembly's length is approximately 90% of the expected one. For these reasons we discarded CABOG and Bambus2.

As shown in Table 5, using Allpaths-LG as master assembly, we were able to increase its NG50 by ~10 Kbp. While GAA behaved better than GAM-NGS in terms of corrected NG50 as its value is ~3Kbp longer, our tool behaved slightly better with consideration of duplication and compression events. Also in this case, ZORRO has worse performance among tested tools in terms of contiguity (both NG50 and corrected NG50). More importantly, Table 6 shows that both GAM-NGS and GAA were able to lower the number of misjoins, while ZORRO introduced a relocation.

**Table 5:** GAGE statistics (contiguity, duplication and compression) on Rhodobacter sphaeroides.

Assembler	Ctg num	NG50 (kb)	NG50 corr. (kb)	Assembly size (%)	Chaff size (%)	Unaligned ref (%)	Unaligned asm (%)	Dupl (%)	Comp (%)
Allpaths-LG	204	42.45	34.42	99.68	0.01	0.45	0.01	0.38	0.31
MSR-CA	395	22.12	19.08	97.02	0.01	3.47	0.04	1.05	0.53

*Allpaths-LG + MSR-CA*									
**GAM-NGS**	168	51.12	37.88	99.97	0.00	0.28	0.01	0.61	0.31
GAA	164	53.82	40.55	100.07	0.01	0.20	0.01	0.63	0.32
ZORRO	216	38.87	30.64	100.41	0.03	0.36	0.02	0.43	0.48

*MSR-CA + Allpaths-LG*									
**GAM-NGS**	199	49.61	37.88	97.95	0.01	3.10	0.04	1.58	0.61
GAA	177	54.71	40.55	99.74	0.01	1.61	0.04	1.08	0.35
ZORRO	206	44.61	38.79	101.14	0.09	0.21	0.06	1.64	0.25

									

Bambus2	177	93.19	12.78	94.97	0.00	4.92	0.01	0.00	0.24
SOAPdenovo	202	131.68	14.34	100.29	0.44	0.76	0.01	1.30	0.46

*Bambus2 + SOAPdenovo*									
**GAM-NGS**	83	149.75	14.16	98.32	0.00	3.02	0.00	1.59	0.63
GAA	100	194.16	14.74	98.35	0.13	2.28	0.01	0.63	0.58
ZORRO	711	16.56	13.18	100.48	0.89	0.66	0.25	1.05	0.59

*SOAPdenovo + Bambus2*									
**GAM-NGS**	177	154.47	15.17	100.41	0.42	0.82	0.01	1.67	0.48
GAA	174	188.18	14.54	100.35	0.44	0.76	0.01	1.38	0.48
ZORRO	720	16.56	12.78	100.48	0.84	0.69	0.24	1.14	0.56

For each assembler we report the number of contigs greater than 200 bp (Ctg), the NG50, the corrected NG50 (NG50 computed breaking the assembly at each error), assembly's total length, the percentage of short (Chaff) contigs, the length of reference's regions which cannot be found in the assembly (Unaligned ref), the length of assembly's regions that cannot be found in the reference (Unaligned asm), the percentage of duplicated (Dupl) and compressed (Comp) regions in the assembly. All the percentages in the table are computed with respect to the true genome size.

**Table 6:** GAGE statistics (SNPs, indels and misjoins) on Rhodobacter sphaeroides.

Assembler	SNPs	Indels < 5 bp	Indels ≥ 5 bp	Misjoins	Inv	Reloc
Allpaths-LG	218	150	37	6	0	6
MSR-CA	807	179	32	9	1	8

*Allpaths-LG + MSR-CA*						
**GAM-NGS**	250	157	44	5	0	5
GAA	345	162	48	5	0	5
ZORRO	263	153	35	7	0	7

*MSR-CA + Allpaths-LG*						
**GAM-NGS**	842	198	46	10	1	9
GAA	802	187	49	10	1	9
ZORRO	928	215	29	7	0	7

						

Bambus2	189	149	363	5	0	5
SOAPdenovo	534	155	404	8	0	8

*Bambus2 + SOAPdenovo*						
**GAM-NGS**	431	173	406	10	0	10
GAA	581	177	404	10	0	10
ZORRO	546	196	84	8	0	8

*SOAPdenovo+ Bambus2*						
**GAM-NGS**	534	153	393	8	0	8
GAA	532	155	407	8	0	8
ZORRO	513	175	111	9	0	9

For each assembly we show the number of SNPs, the number of indels shorter than 5 bp and greater (or equal) than 5 bp. The number of misjoins is computed as the sum of inversions (parts of contigs reversed with respect to the reference genome) and relocations (rearrangements moving a contig within/between chromosomes).

When using MSR-CA as master assembly, GAM-NGS was able to increase MSR-CA's NG50 by ~27 Kbp, providing a longer corrected NG50 with respect to the two merged assemblies. Also with this master/slave combination, GAA's assembly is characterized by a corrected NG50 slightly better than GAM-NGS's one. Both GAM-NGS and GAA introduced one additional misjoin with respect to MSR-CA, while ZORRO was able to correct the master assembly.

Table 5 and Table 6 also show the results of merging the assemblies with the highest NG50 (*i.e*., Bambus2 and SOAPdenovo). GAM-NGS and GAA have very similar statistics and for both of them the difference between the NG50 and its corrected value is substantial. ZORRO, instead, tends to output a highly fragmented assembly lowering the number of indels but without correcting any misjoin.

Table 7 shows running times of the three assembly reconciliation tools. Also in this dataset, if we consider the CPU time, then GAM-NGS is definitely affected by the required reads alignment phase and requires much more time than GAA and ZORRO. If we consider wall time, instead, GAM-NGS runs in less than 8 minutes, comparable, if not better, than the other tools.

**Table 7:** Assembly reconciliation tools performances on Rhodobacter sphaeroides.

Tool	User (CPU) time	Wall clock time
*Allpaths-LG + MSR-CA*		
**GAM-NGS**	1h 21' 09" + 2' 20"	5' 03" + 43"
GAA	17"	17"
ZORRO	14' 46"	14' 46"

*MSR-CA + Allpaths-LG*		
**GAM-NGS**	1h 21' 09" + 2' 19"	5' 03" + 48"
GAA	19"	19"
ZORRO	16' 15"	16' 15"

*Bambus2 + SOAPdenovo*		
**GAM-NGS**	1h 26' 47" + 2' 35"	5' 53" + 1' 13"
GAA	3' 59"	3' 59"
ZORRO	8' 22"	8' 22'

*SOAPdenovo + Bambus2*		
**GAM-NGS**	1h 26' 47" + 2' 23"	5' 53" + 1' 09"
GAA	3' 47"	3' 47"
ZORRO	7' 44"	7' 44"

In GAM-NGS's entries the first value indicates the time spent in alignment phase, while the second one is GAM-NGS's run time.

#### Human chromosome 14

These first two bacteria datasets are small and time might not be considered an issue (each assembly reconciliation tool was able to run in reasonable time). The third GAGE dataset on which we tested our tool was the human chromosome 14 (characterized by an ungapped 88 Mbp size). This dataset is not only ~20 times larger than the other two, but it is also more complex (*e.g*., containing repeats, afflicted by heterozygosity). Moreover, in this scenario GAM-NGS starts to show its real potential: assembling large datasets using a relatively low amount of resources, while preserving correctness.

ZORRO output is not shown in Table 8 as, after two weeks of computation, it was not able to provide an output. Thus, we limit our evaluation to only GAM-NGS and GAA.

**Table 8:** GAGE statistics (contiguity, duplication and compression) on human chromosome 14.

Assembler	Ctg num	NG50 (kb)	NG50 corr. (kb)	Assembly size (%)	Chaff size (%)	Unaligned ref (%)	Unaligned asm (%)	Dupl (%)	Comp (%)
Allpaths-LG	4529	27.96	15.69	78.67	0.02	20.03	0.04	0.23	2.11
CABOG	3361	35.86	18.63	80.34	0.02	19.13	0.07	0.13	1.39

*Allpaths-LG + CABOG*									
**GAM-NGS**	2235	61.64	21.91	80.94	0.02	19.08	0.10	0.88	1.43
GAA	1989	69.40	23.04	82.08	0.02	18.92	0.09	1.52	1.39

*CABOG + Allpaths-LG*									
**GAM-NGS**	1979	66.29	23.63	81.00	0.02	19.00	0.06	0.74	1.37
GAA	1903	70.39	23.89	81.89	0.02	18.98	0.07	1.21	1.36

For each assembler we report the number of contigs greater than 200 bp (Ctg), the NG50, the corrected NG50 (NG50 computed breaking the assembly at each error), assembly's total length, the percentage of short (Chaff) contigs, the length of reference's regions which cannot be found in the assembly (Unaligned ref), the length of assembly's regions that cannot be found in the reference (Unaligned asm), the percentage of duplicated (Dupl) and compressed (Comp) regions in the assembly. All the statistics were computed using the same script with the gapped reference genome (107,349,540 bp).

**Figure 8:**
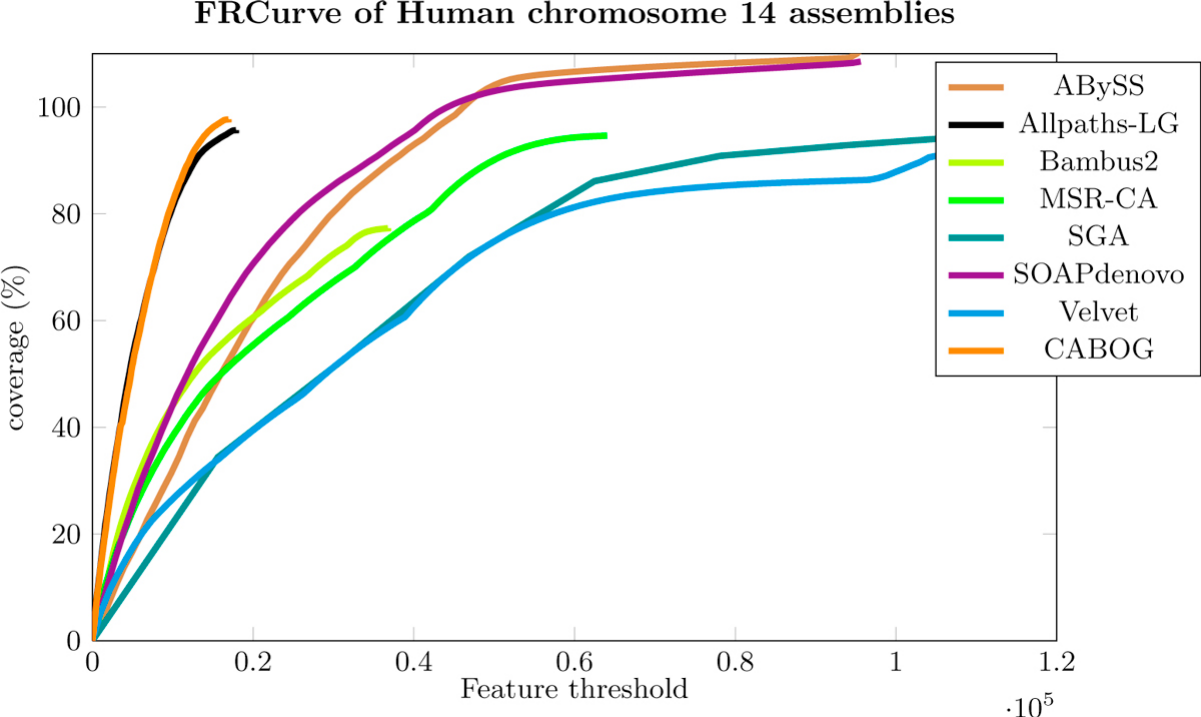
**FRCurve of Human chromosome 14 assemblies**. FRCurve of human chromosome 14. Allpaths-LG and CABOG contain definitely the lowest numbers of features with respect to the other assemblers.

For this dataset we chose to merge Allpaths-LG and CABOG assemblies. Looking at their FRCurves in Figure 8, they are clearly the best two assemblies to be merged. GAGE's statistics also show that Allpaths-LG and CABOG assemblers produce the best two assemblies for this dataset (*i.e*., highest NG50 and low number of misjoins).

Table 9 shows how, using Allpaths-LG as master assembly, GAM-NGS was able to increase NG50 by ~ 32 Kbp and the corrected NG50 by ~ 6 Kbp. GAA returned better NG50 values but it produced more duplicated regions and it was afflicted by a larger amount of misjoins and indels compared to GAM-NGS.

**Table 9:** GAGE statistics (SNPs, indels and misjoins) on human chromosome 14.

Assembler	SNPs	Indels < 5 bp	Indels ≥ 5 bp	Misjoins	Inv	Reloc
Allpaths-LG	55319	27563	2558	101	44	57
CABOG	81151	28438	2884	149	46	103

*Allpaths-LG + CABOG*						
**GAM-NGS**	61725	29936	2950	119	32	87
GAA	63835	30151	2990	123	29	94

*CABOG + Allpaths*						
**GAM-NGS**	79478	29653	3021	154	43	111
GAA	81763	29812	3008	134	31	103

For each assembly we show the number of SNPs, the number of indels shorter than 5 bp and greater (or equal) than 5 bp. The number of misjoins is computed as the sum of inversions (parts of contigs reversed with respect to the reference genome) and relocations (rearrangements moving a contig within/between chromosomes). All the statistics were computed using the same script with the gapped reference genome (107,349,540 bp).

**Figure 9:**
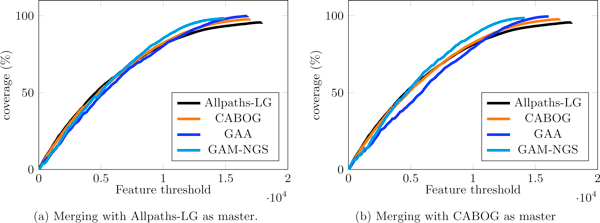
**FRCurve of assembly reconciliation tools**. FRCurve of assembly reconciliation tools on human chromosome 14, using (a) Allpaths-LG and (b) CABOG as master assembly. Despite the lower corrected NG50 (which means errors in the longest contigs), considering the whole assembly, GAM-NGS seems to behave globally better than GAA and the input assemblies.

We also want to point out that the corrected NG50 is certainly an important statistic to evaluate the improvement of a merge with respect to the master assembly but it only indicates whether the longest contigs are affected by errors and does not tell how the assembler behaves on short contigs (which are also important to assess assemblies' quality, as FRCurve demonstrates). We finally plot the FRCurve to globally estimate the quality of the merged assemblies. Figure 9 shows that GAM-NGS globally behaved better and, in particular, seems to introduce less features (especially in the shortest contigs of the assembly).

Table 10 shows running times of the two assembly reconciliation tools used with this dataset. GAM-NGS required about 1 hour to accomplish its task (reads' alignments included), while GAA required about 13 hours (manually running multiple BLAT alignments in parallel).

**Table 10:** Assembly reconciliation tools performances on human chromosome 14.

Tool	User (CPU) time	Wall clock time
*Allpaths-LG + CABOG*		
**GAM-NGS**	4h 24' 59" + 1h 14' 41"	45' 56" + 18' 16"
GAA	452h 18'	14h 16' 4"

*CABOG + Allpaths-LG*		
**GAM-NGS**	4h 24' 59" + 1h 12' 35"	45' 56" + 19' 21"
GAA	467h 40'	13h 44' 58'

In GAM-NGS's entries the first value indicates the time spent in alignment phase, while the second one is GAM-NGS's run time. Due to the size of the assemblies, we parallelized BLAT's execution to get GAA's output in a reasonable time. ZORRO results are not shown due to the fact that the tool cannot run in parallel and, after more than a week of computation, was still not able to provide an output.

This characteristic may not be very important for short genomes but, as the size increases, it becomes of crucial importance. As we will show in the tests on some large plant genomes, GAM-NGS is able to merge even 20 Gbp assemblies using a relatively low amount of memory and time.

### GAM-NGS's performances on large datasets

On small datasets, all the assembly reconciliation tools provide an output in reasonable time. However, when we consider the human chromosome 14 we observe how GAA runs at least 10 times slower than GAM-NGS (if we consider also the mandatory reads' alignment step) while ZORRO, after two weeks, is not even able to provide us a partial output. This proves that the major bottleneck consists in the global alignment phase of these tools.

On the contrary, GAM-NGS's approximation (using read's alignment back to the assemblies) coupled with the implementation of a weighted graph, achieves similar results in a reasonable amount of time. In order to show GAM-NGS's scalability, we tested it on three large plants genomes whose sizes vary from 227 Mbp to 20 Gbp.

The first of these datasets is *Prunus persica*, characterized by a genome size of 227 Mbp. The best assemblies we were able to compute were produced with CLC and ABySS assemblers, which were similar in length and number of contigs. We chose to use ABySS as master, since it was more contiguous. As shown in Table 11, we were able to increase NG50 (of ~3 Kbp with respect to the master) and provide a more contiguous assembly compared to both CLC and ABySS. After mapping a 65× coverage of Illumina paired-end reads (which required 4 hours and 37 minutes), GAM-NGS took less than 2 hours using at most 19.6 GB of RAM.

**Table 11:** GAM-NGS's contiguity statistics on large plants datasets

Assembler	Total Length (Kbp)	Contigs	N50 (bp)
*Prunus persica*			
ABySS (M)	177,460	33,949	10,895
CLC (S)	179,151	41,684	8,654
**GAM-NGS**	**184,735**	**27,445**	**13,410**
*Populus nigra*			
CLC (M)	339,551	104,432	6,130
ABySS (S)	296,245	83,564	5,357
**GAM-NGS**	**359,795**	**78,366**	**10,018**

The second large dataset we used is *Populus nigra*, characterized by a genome size of ~423 Mbp. Also in this case, as for *Prunus persica*, the assemblies we had at our disposal were made with CLC and ABySS. This time, CLC's assembler looked better for its total length and NG50 and, thus, we decided to use it as master. As shown in Table 11, even with this dataset, we were able to increase NG50 (by ~4 Kbp with respect to the master) and to provide a more contiguous assembly. To perform the mandatory alignment step we used a 80× coverage of Illumina paired-end reads, which required about 8 hours. Then, GAM-NGS took less than 4 hours using at most 34.5 GB of RAM to perform the merge. In order to save memory we could have decreased the reads coverage (at least 30× is suggested at the cost of a lower assembly improvement).

As a demonstration of GAM-NGS's flexibility, consider that GAM-NGS has also been used to obtain an assembly of the 20 Gbp genome of *Picea abies*, where performing a global alignment is impracticable.

In this scenario, the idea was to improve a Whole Genome Shotgun (WGS) assembly $A_{WGS}$ with a set of fosmid pools FP  sampled from the same genome. Each fosmid pool was sequenced and assembled separately using a 80× coverage. Then, the 50× coverage of Illumina reads used to assemble $A_{WGS}$ has been mapped on both $A_{WGS}$ and FP  for the blocks construction phase. GAM-NGS was able to run in less than 3 days (6 days, taking into account also the mandatory alignment phase) using at most 612 GB of RAM. These is certainly a low amount of resources, considering the dataset's size (almost a Terabyte) and that building the WGS assembly took one week and required more than 1 TB of RAM. Furthermore, GAM-NGS was able to increase the assembly length by 1.4 Gbp of the estimated genome size with a NG50 that was 1.42 times greater than the one of the WGS assembly (data not yet published and not yet publicly available, we were allowed to show only the increment of the statistics with respect to the assembly we wanted to improve).

## Conclusions and future work

GAM-NGS is a *de novo *graph-based assembler which is able to merge assemblies using a (relatively) low amount of computational resources. Its strength relies on the fact that it does not need a global alignment to be performed and that makes our strategy unique among the other assembly reconciliation tools. In fact, GAM-NGS finds regions belonging to the same DNA *locus *using reads aligned back to the assembly, which is an almost mandatory analysis step in all *de novo *assembly projects. The order in which these regions have been assembled is exploited to build a locally weighted graph that GAM-NGS uses to fill gaps between sequences and to correct putative mis-assemblies. Moreover, mapping reads to the assemblies (thus, without knowing how they have been placed by the assemblers) may lead to complex graph sub-structures (*e.g*., bubbles, bifurcations, cycles) due to alignment errors or chimeric assembly sequences. Resolving these types of sub-graphs is not a trivial task, as in certain regions there may be lack of any possible evidence. In these kind of situations (which, for instance, represented 40% of the problematic cases for the human GAGE's dataset) we decided to be as conservative as possible, returning the sequences of one of the assemblies (elected as master by the user).

In this paper we validated our tool using GAGE [10] datasets, proving its effectiveness and reliability. Results showed that, for each GAGE dataset, GAM-NGS was always able to improve master assembly's NG50 and corrected NG50 (*i.e*., NG50 of the assembly broken in correspondence of the errors), thus providing a globally more correct output (even if some errors were carried by the slave assembly). Although GAA provided better statistics in some cases, GAM-NGS gives comparable results and offers excellent scalability. GAM-NGS yields an improved assembly in reasonable time on large datasets (especially if used on a multicore computer) for which competing tools are impractical. In particular, we showed GAM-NGS's scalability on large (plant) datasets (genome size up to 20 Gbp), where our tool required a low amount of computational resources compared to the dataset sizes and assembly requirements.

The presented algorithm performs a merge of two assemblies, returning the sequences of one of them in those problematic regions where we are not able to determine the most correct sequence between the two assemblies. We plan to investigate the use of further weights in AG that will allow us to solve more "difficult" regions, allowing us to completely replace the master-slave approach with a strategy that provides a more correct output.

We also plan to exploit GAM-NGS in a strategy thought to improve and correct a Whole Genome Shotgun assembly along with multiple sets of well assembled fosmid (or BAC) pools which constitute a hierarchically simplified version of the same genome.

## Availability and requirements

GAM-NGS's source can be freely downloaded from http://github.com/vice87/gam-ngs. It has been written in C++ and has been tested on Linux operating systems.

## Abbreviations

NGS: Next Generation Sequencing; AG: Assemblies Graph; CG: Contigs Graph; WGS: Whole Genome Shotgun.

## Competing interests

The authors declare that they have no competing interests.

## Authors' contributions

RV, FV, SS, LA and AP equally contributed to the idea and to the design of the algorithm and the experiments. RV developed the tool. RV and SS performed the experiments. RV, FV, SS, LA and AP wrote the paper.

## Supplementary Material

Additional file 1 **Commands run in the experiments**. A brief description of the commands we used to carry out the merging on all the datasets. Click here for file

## Declarations

The publication costs for this article were supported by Epigenomics Flagship Project (Progetto Bandiera Epigenomica) EPIGEN.

This article has been published as part of *BMC Bioinformatics *Volume 14 Supplement 7, 2013: **Italian Society of Bioinformatics (BITS): Annual Meeting 2012**. The full contents of the supplement are available online at http://www.biomedcentral.com/bmcbioinformatics/supplements/14/S7
