# Supplementary material for: GAM-NGS: genomic assemblies merger for next generation sequencing
Source: BMC Bioinformatics. 2013 Apr 22;14(Suppl 7):S6. doi: 10.1186/1471-2105-14-S7-S6 (PMC3633056; doi:10.1186/1471-2105-14-S7-S6)
Supplement: Additional file 1 — Commands run in the experiments. A brief description of the commands we used to carry out the merging on all the datasets. [file 1471-2105-14-S7-S6-S1.pdf]

GAM-NGS: Genomic Assemblies Merger for Next Generation Sequencing

Riccardo Vicedomini, Francesco Vezzi, Simone Scalabrin,
Lars Arvestad, Alberto Policriti

Commands run in the experiments

We ran **GAA** (version 1.1) using the following commands:

```
$ blat assembly1.fasta assembly2.fasta out.psl;
$ perl gaa.pl --target assembly1.fasta --query assembly2.fasta --match out.psl
```

We had to manually run BLAT command because, internally to GAA, it is called with the option `-fastMap`, which works only if all query's contigs are shorter than 5 Kb.

We ran **ZORRO** (version 2.2) using the following command:

```
$ perl zorro.pl --assembly1 assembly1.fasta --assembly2 assembly2.fasta
                --readset frags.fasta
```

where `frags.fasta` is a FASTA file containing a subset of dataset's reads (about 10× of coverage).

Finally, we ran **GAM-NGS** using the following commands:

```
$ ./gam-create --master-bam master.PE.align.bams.txt
               --slave-bam slave.PE.align.bams.txt
               --min-block-size B_min --output output;

$ ./gam-merge  --master-bam master.PE.align.bams.txt
               --slave-bam slave.PE.align.bams.txt
               --master-mp-bam master.MP.align.bams.txt
               --slave-mp-bam slave.MP.align.bams.txt
               --master-fasta master.fasta --slave-fasta slave.fasta
               --blocks-file output.blocks --min-block-size B_min
               --coverage-filter T_c --threads THREADS_NUM --output output
```
